# Evaluation of the use of a guided bur during preclinical teaching of tooth preparation: A pilot study

**DOI:** 10.1002/cre2.184

**Published:** 2019-09-30

**Authors:** Soho Yee, Raphaël Richert, Gilbert Viguie, Sébastien Couraud, Marion Dehurtevent, Michel Fages, Pascale Corne, Maxime Ducret

**Affiliations:** ^1^ Faculté d'Odontologie Université de Lyon, Université Lyon 1 Lyon France; ^2^ Hospices Civils de Lyon Service de Consultations et Traitements Dentaires Lyon France; ^3^ Faculté de médecine et de maïeutique Lyon‐Sud Université de Lyon, Université Lyon 1 Lyon France; ^4^ Service de Pneumologie Aiguë Spécialisée et Cancérologie Thoracique Centre Hospitalier Lyon Sud Pierre Bénite France; ^5^ Faculté d'Odontologie Université de Lille Lille France; ^6^ Prosthetic Department Faculté d'Odontologie de Montpellier Montpellier France; ^7^ Laboratoire de Bioingéniérie et Nanosciences (LBN) Montpellier Université Montpellier France; ^8^ Nancy School of Dentistry University of Lorraine Nancy Cedex France; ^9^ Laboratoire de Biologie Tissulaire et Ingénierie thérapeutique UMR5305 CNRS/Université Lyon 1, UMS3444 BioSciences Gerland‐Lyon Sud Lyon France

**Keywords:** dental education, prosthodontics, technique skills, virtual assessment software

## Abstract

**Objectives:**

An innovative calibrated bur, aiming to improve precision during reduction of the incisal edge, was recently proposed to guide practitioners during tooth preparation. However, limited information is available concerning its usefulness in dental preclinical education. The aim of this study was to evaluate whether using this innovative guided bur improves learning experience quality and the performance of students during tooth preparation.

**Material and methods:**

After having provided written consent, 60 second‐year students were divided into two groups. One group used a 1‐mm rounded bur to perform depth grooves, whereas the second group used the innovative guided bur, consisting in a 2‐mm‐depth marker with a stopping surface. Once the grooves were obtained, they were then connected using the same wheel bur in both groups. The aim was to obtain a final 2‐mm reduction of the incisal edge. Quality of the learning experience (stress level, motivation to restart, self‐evaluation of the preparation, and difficulty) was quantified using a visual analog scale. Duration of the procedure was also measured in both groups. 3D measurements for each tooth were performed using an STL comparison software.

**Results:**

There were no significant differences between groups in terms of stress and self‐evaluation of the preparation. Students in the guided bur group reported significantly lower perception of exercise difficulty (*p* < .001) and significantly higher motivation to restart the procedure (*p* < .001). The guided bur group performed the procedure in 16.4% less time than the rounded bur group. The use of the guided bur led to a 23% over‐reduction, whereas the use of the rounded bur led to a 10% under‐reduction.

**Conclusions:**

Overall, the present study shows that the guided bur provides significant improvement in the student's learning experience with increased motivation and decreased perception of difficulty. It shortens the duration of procedure performance, but it also induces a reduction in preparation accuracy.

## 1 INTRODUCTION

Preclinical training in prosthetic dentistry is generally focused on acquiring knowledge, manual dexterity, and technical skills (Clancy, Lindquist, Palik, & Johnson, 2002). Acquisition of prosthetic psychomotor skills therefore requires regular practice in a wide range of clinical situations. However, due to the intensive nature of dental courses and limited school resources, student learning time is often restricted. An important aspect of training is the ability to visualize simultaneously all prosthetic parameters while performing the procedure (Güth et al., 2013; Habib, 2018; Mays & Branch‐Mays, 2016). This is of particular importance for crown preparation. It is indeed reported that insufficient incisal reduction is one of the most frequent problems encountered during training (Christensen, 2007; Syed, Al‐Moaleem, & Shariff, 2016). A recent strategy to improve quality of training proposes to concentrate on individual tasks, during initial learning, in order to reduce attention demands and increase knowledge recall (Winning, Malhotra, & Masters, 2018).

The evaluation of innovative learning strategies is well established and comprises several aspects, from technical performance (behavior evaluation) to learning experience and environment (Bates, 2004). Performance requires the use of scales and instruments to examine a range of variables during training (Bates, 2004). The learning experience is typically gauged through the learner's reactions, by assessing factors such as interest, motivation, difficulty, and attention levels (Bates, 2004). A positive learning experience is important for the well‐being, academic achievement, and success of students (Brown, Williams, & Lynch, 2011; Hutchinson, 2003; Stormon, Ford, & Eley, 2018; Tiu et al., 2016). However, this has yet to be investigated in the field of prosthodontics preclinical training.

In 1977, Preston developed a “guided” technique to improve tooth preparation. The first step consisted in making depth grooves using a rounded diamond bur to then remove the portions between the orientation grooves. More recently, studies have reported that the use of guided burs may represent a valuable option to help dental students and dentists during tooth preparation (Fages & Bennasar, 2013; Fages, Bennasar, & Raynal, 2017). One study in particular reported that guided burs improve the quality of occlusal reduction, when performed by sixth‐year dental students (Rosella et al., 2015). However, the authors of the study used different burs in a complex multitask procedure that could lead to cognitive overload when used with younger preclinical students. Indeed, performance can be disrupted if demands are increased by multitasking (Winning et al., 2018). Moreover, quality of occlusal reduction was assessed based on the visual evaluation without the use of a virtual tool that would have rendered the results more objective and accurate (Esser, Kerschbaum, Winkelmann, Krage, & Faber, 2006; Sadid‐Zadeh & Feigenbaum, 2018; Tiu et al., 2016). The present investigation proposes to overcome the limitation of visual evaluation by using a standardized computer‐based approach and to limit the cognitive overload by using a simplified procedure.

The aim of the study was to evaluate the impact of the guided bur on quality of learning experience and student performance (accuracy in reduction of the incisal edge; time for procedure performance) when comparing it with the use of the rounded bur during preclinical training of tooth preparation.

## 2 MATERIAL AND METHODS

### 2.1 Study sample selection

Ethical approval was obtained from the local ethics committee (Comité éthique du CHU de Lyon, reference number 18‐01). The study was conducted in 2018 in the Lyon teaching hospitals (Hospices Civils de Lyon, France). Sixty voluntary participants were selected from a pool of second‐year dental students of the Dental Faculty (Université Claude Bernard Lyon 1, France). All participants provided written informed consent after having received information about the study. These students had received theoretical instruction but had no experience in preparing anterior ceramic crowns. Participants were randomly divided into two groups of equal size. The first group, defined as the control group, used Preston's technique (rounded bur with adequate protocol). The second group, or test group, received the guided bur protocol. Students were allowed to ask any questions concerning procedure details before starting the study.

### 2.2 Incisal reduction

**Figure 1:**
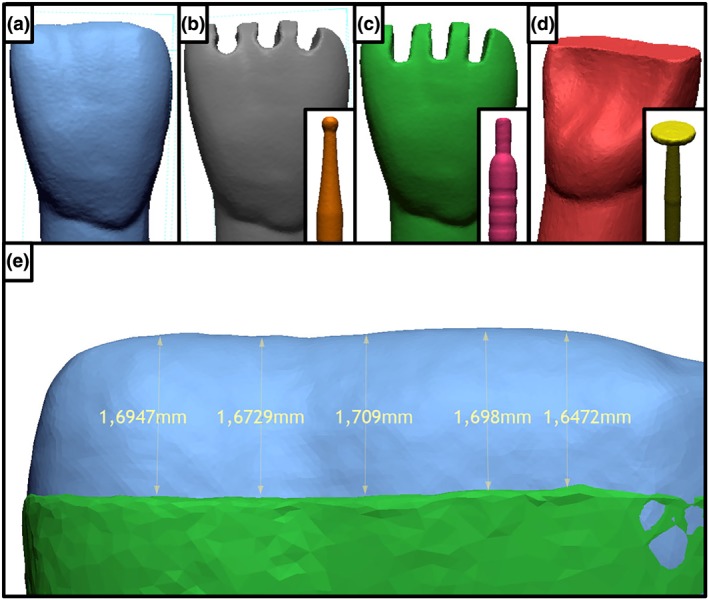
Protocol for incisal reduction and measurement. (a) Initial model; (b) grooves prepared twice using diamond rounded burs (1 mm in diameter); (c) grooves prepared using calibrated burs (2 mm in height); (d) model prepared after connecting the grooves using a wheel bur; and (e) depth of the preparation measured manually using digital tools, after matching to reference model. Please note that the presented model is under‐reduced

The goal of the present procedure was to achieve a 2‐mm incisal reduction, in the axial direction, on artificial #11 typodont tooth (Figure 1a; tooth ref no. 0.63.1115, Kavo Dental, Lognes, France). First, the incisal reduction was initiated by performing three orientation grooves. The control group used a 1‐mm rounded bur (ref no. 6801 314 010, Komet Dental, Lemgo, Germany) (Figure 1b), whereas the test group used a specific 2.0‐mm‐long and 0.9‐mm‐diameter guided bur with a stopping surface (ref no. MADC 20, NTi, Kahla, Germany; Figure 1c). The next step was to connect these grooves using a wheel bur (ref no. 818 040C‐FG, NTi) to obtain a 2‐mm calibrated reduction of the central incisal edge (Figure 1d).

### 2.3 Method of measurement

Quality of the learning experience was quantified by each student answering four questions using a visual analog scale. The visual analog scale consisted in a straight horizontal line defined between 0 and 10 (0 being the lowest level of appreciation reported and 10 the highest). Questions included (a) determining levels of stress prior to performing the procedure (b) assessing motivation to restart the procedure, (c) visually self‐evaluating their own preparation, and (d) assessing the level of difficulty of the procedure. Visual analog scales were converted in numerical values and rounded to their nearest mid point, using a graduated ruler that limits the risk of mistakes during the process. Execution time for the procedure was recorded using the student's phone chronometers. An unprepared reference tooth was scanned using a laboratory scanner (LabScan HD®, Bego France, Villeurbanne, France). All the prepared teeth were scanned by intraoral scanner (Trios 3®; 3Shape, Copenhagen, Denmark) in STL format. These files were blinded before analysis. STL files for each prepared tooth were then superimposed to the reference STL of the unprepared tooth, using automatic matching algorithm of the software (Geomagic®, Design X, 3D Systems). Fifteen 3D measurements for each tooth were performed using an STL comparison software (Geomagic®, Design X, 3D Systems; Figure 1e).

### 2.4 Data collection and statistical analysis

Questionnaires were collected, and visual analog scales were converted to a numerical value on a scale of 10. The 3D STL measurements were then compiled and blinding lifted. All quantitative data were verified for normal distribution using the Shapiro–Wilk test (*α* = .05). All data are expressed as median, interquartile range [IQR] in grades/10, seconds, or millimeters where appropriate. Data are presented as box plots (GraphPad Prism 7, GraphPad, La Jolla, CA, USA). Data were not normally distributed, and a Wilcoxon test was applied. Differences between groups were considered statistically significant for *p* ˂ .05.

## 3 RESULTS

### 3.1 Learning experience

**Figure 2:**
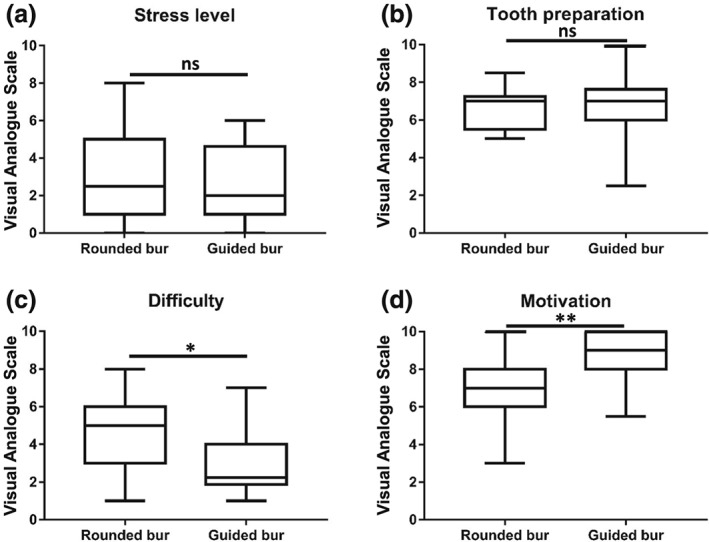
Box plots representing quality of the learning environment (*n* = 30; ^*^ *p* < .05; ^**^ *p* < .001; ns, not significant)

Self‐reported stress, evaluated on a 10‐point scale, was not significantly different between the guided bur group (median: 2.5, IQR[1–4,7]) and the rounded bur group (median: 2, IQR[1,1–5]; *p* = .29; Figure 2a). Self‐evaluation of tooth preparation was also similar for the group using the guided bur (median: 7, IQR[6–7,6]) when compared with the control group (median: 7, IQR[5,6–7,2]; *p* = .99; Figure 2b). Students reported significantly lower perception of exercise difficulty when using the guided bur (median: 2.25, IQR[1,9–4]) rather than the rounded bur (median: 5, IQR[3–6]; *p* < .001; Figure 2c). Students also reported significantly greater motivation to restart the procedure in the guided bur group (median: 9, IQR[8–10]) compared with the control group using the rounded bur (median: 7, IQR[6–8]; *p* < .001; Figure 2d).

### 3.2 Behavior evaluation

**Figure 3:**
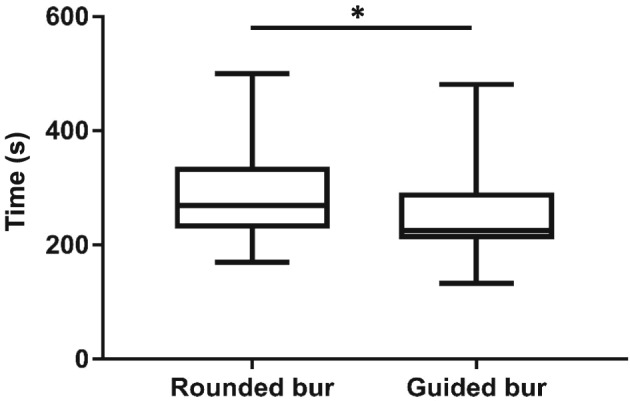
Box plots representing time for procedure performance (*n* = 30; ^*^ *p* < .05)

**Figure 4:**
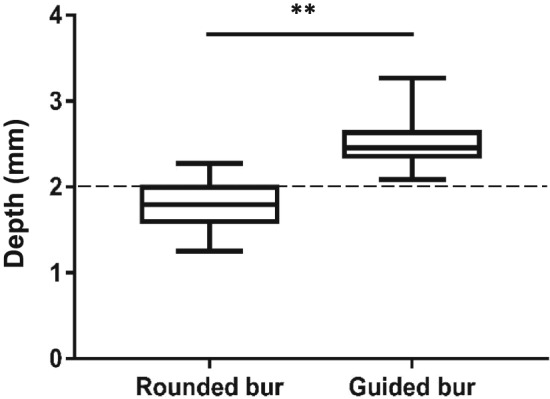
Box plots depicting depth obtained after incisal reduction (*n* = 30; ^**^ *p* < .001)

Procedure duration time (s) was significantly shorter for the test group (median: 225, IQR[215–287]), compared with the control group (median: 269, IQR[234–333]; *p* < .05; Figure 3). Preparation depth measured at the end of the procedure was significantly greater when using guided burs (median: 2.47, IQR[2,33–2,66]), compared with rounded burs (median: 1.77, IQR[1,60–1,99]; *p* < .001; Figure 4). In terms of the 2‐mm incisal reduction goal, the guided bur led to a mean of 23% over‐preparation and the rounded bur to a mean of 10% under‐preparation.

## 4 DISCUSSION

For many decades, authors have suggested to start tooth preparation using depth orientation grooves performed with a rounded tapered diamond bur. Recently, an innovative procedure using guided burs was proposed. In the present study, we report that the use of this guided bur provides significant improvement in the student's learning experience (motivation and perception of difficulty) and shortens the duration of the procedure. However, this technique also appears to induce a reduction in accuracy during tooth preparation.

Students are regularly asked to complete a detailed evaluation of the teaching program and teaching effectiveness using questionnaires. Evaluation of the learning experience is less frequent although it is a crucial element for the well‐being and success of students (Brown et al., 2011). The present study failed to find a significant difference in student self‐evaluation of tooth preparation, although significant differences were found in terms of preparation depth. The difficulty in self‐evaluating one's work is strongly associated with lack of knowledge, practical skills, and clinical experience (Tuncer, Arhun, Yamanel, Çelik, & Dayangaç, 2015). Herein, participants were second‐year students with a low level of knowledge or experience in tooth preparation. To increase accuracy in self‐evaluation of dental preparations, various authors have proposed the use of digital tools. However, there is no clear consensus yet as to whether or not these truly improve the self‐assessment capacities of students (Esser et al., 2006; Gratton, Kwon, Blanchette, & Aquilino, 2016). Moreover, some authors have also suggested that self‐assessment can be linked to stress levels (Pope, 2005). Stress evaluation is difficult to investigate in education due to inter‐individual variability in levels of stress, often linked to the grading systems (Alzahem, Van Der Molen, Alaujan, Schmidt, & Zamakhshary, 2011; Elani et al., 2014; Pöhlmann, Jonas, Ruf, & Harzer, 2005; Shah, Hasan, Malik, & Sreeramareddy, 2010; Stormon et al., 2018). In the present study, tooth preparation performance did not count towards the end of year grades. This likely explains the low levels of stress perceived by students in both groups prior to performing the procedure.

The use of guided burs, however, significantly reduced the perceived difficulty in performing the procedure and increased the students' motivation to restart the procedure. These results are in line with a previous study reporting that when a task difficulty increases, student motivation decreases (Lynch, Patten, & Hennessy, 2013). Interestingly, it has also been shown that the introduction of innovative teaching strategies are major components for increasing student motivation and performance (Wery & Thomson, 2013).

In a preclinical situation, behavior and performance are the main criteria to evaluate the trainees' ability to use their knowledge or skills in the workplace. The time reduction in procedure performance induced by the use of guided burs is of particular interest as studies have highlighted the need to find strategies to increase time and cost efficiency in the teaching environment (Serdyukov, 2017). Furthermore, the use of digital tools may also reduce the time needed for evaluation, while providing an objective and standardized method (Callan, Palladino, Furness, Bundy, & Ange, 2014; Esser et al., 2006; Gratton et al., 2016; Güth et al., 2013; Marghalani, 2016). However, assessment by teaching staff and the use of personal feedback remain essential for students to improve (Chambers & Labarre, 2014; Davis et al., 2006).

The results of this study also showed that the guided bur leads to an over‐preparation and the rounded bur to an under‐preparation, with the latter being closer to the goal given. Thus, the rounded bur could be considered more precise. This is important as it impacts the clinical prognostic for restoration. Indeed, under‐preparation limits the space needed for restoration material, whereas over‐preparation likely weakens the remaining tooth structure. However, the impact of the wheel bur on the final reduction depth was not anticipated in this study, and intermediate measurement of groove reduction could have been performed. The choice of the wheel bur used for connecting the grooves therefore needs to be taken into account. Further studies are thus needed to improve and optimize bur selection for tooth reduction procedures. Overall, this innovative guided bur may be of interest in preclinical but also in clinical practice (Schlichting, Maia, Baratieri, & Magne, 2011), especially for posterior teeth preparation, as grooves are more complicated to obtain and depth harder to estimate (Fages et al., 2017; Fages & Bennasar, 2013; Rosella et al., 2015). Importantly, it has been reported that including guided burs during tooth preparation renders the procedure quick and simple (Fages et al., 2017).

## 5 CONCLUSION

The guided bur is of interest as it provides significant improvement in the learning experience and reduces duration of procedure performance by second‐year students. This technique was however linked to a reduction in accuracy that may be related to the type of bur chosen. Further investigation is thus required to optimize bur selection for preclinical training in prosthetic dentistry.

## CONFLICT OF INTEREST

The authors report no conflict of interest.
